# Boracite

**Published:** 1880-08

**Authors:** 


### BORACITE.

SOME years ago, a pamphlet was published by Becker on "*Boracite, the secret remedy of Paracelsus for stone*," (Mühlhausen, 1868). Koehler, taking the suggestion from this, experimented with the remedy with, as he considers, favorable results. In preference to boracite (the borate of magnesium), Koehler used a combination of it with citric acid, in which it is readily soluble, forming boro-citrate of magnesium. In five cases of gravel in which it was tried it seemed to promote the discharge of "sediment or small stones, and afterward to keep the urine clear." A small stone, about half as large as a pea, was immersed in a solution of the salt, and within eight days was crumbled and reduced to a fine sand.

